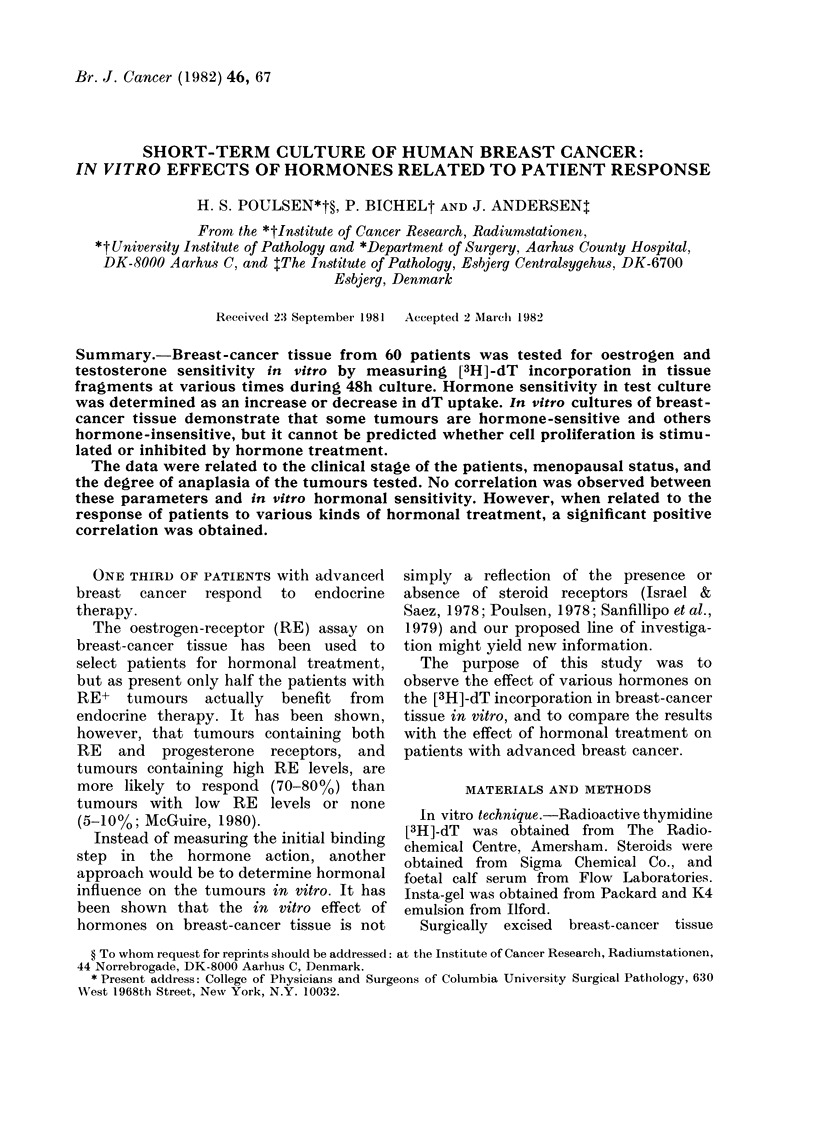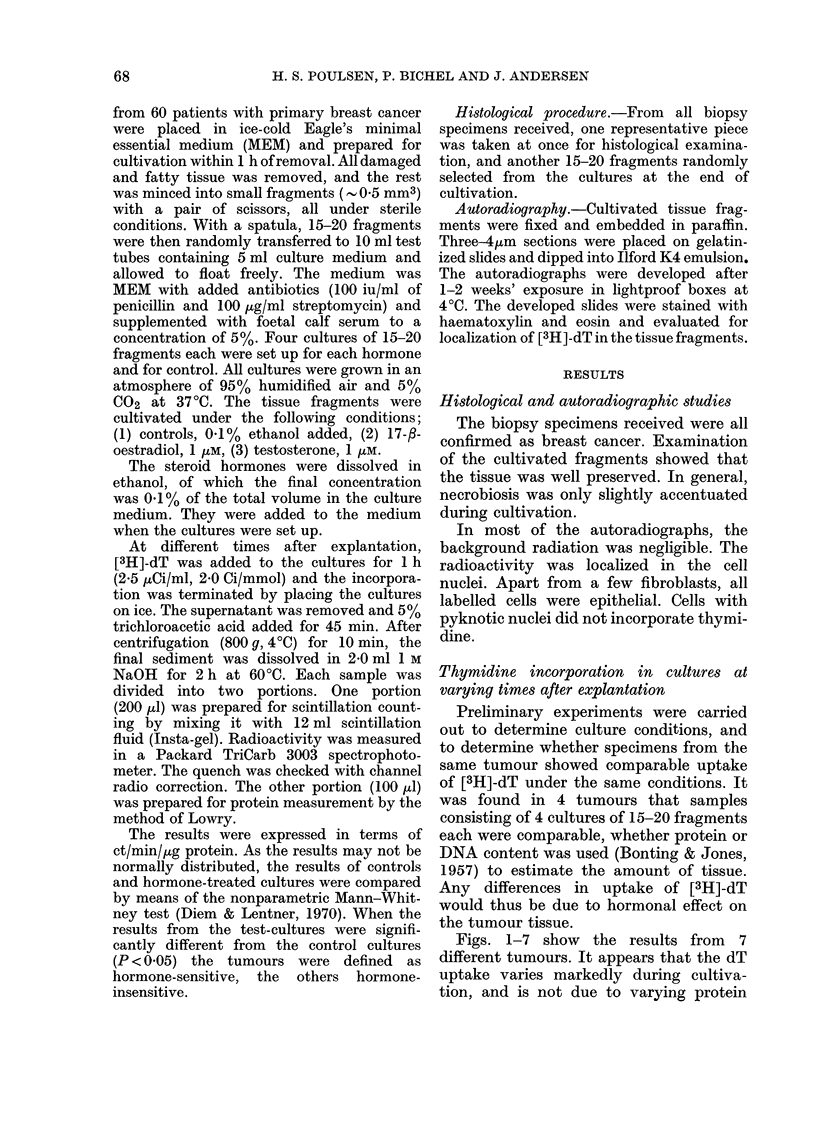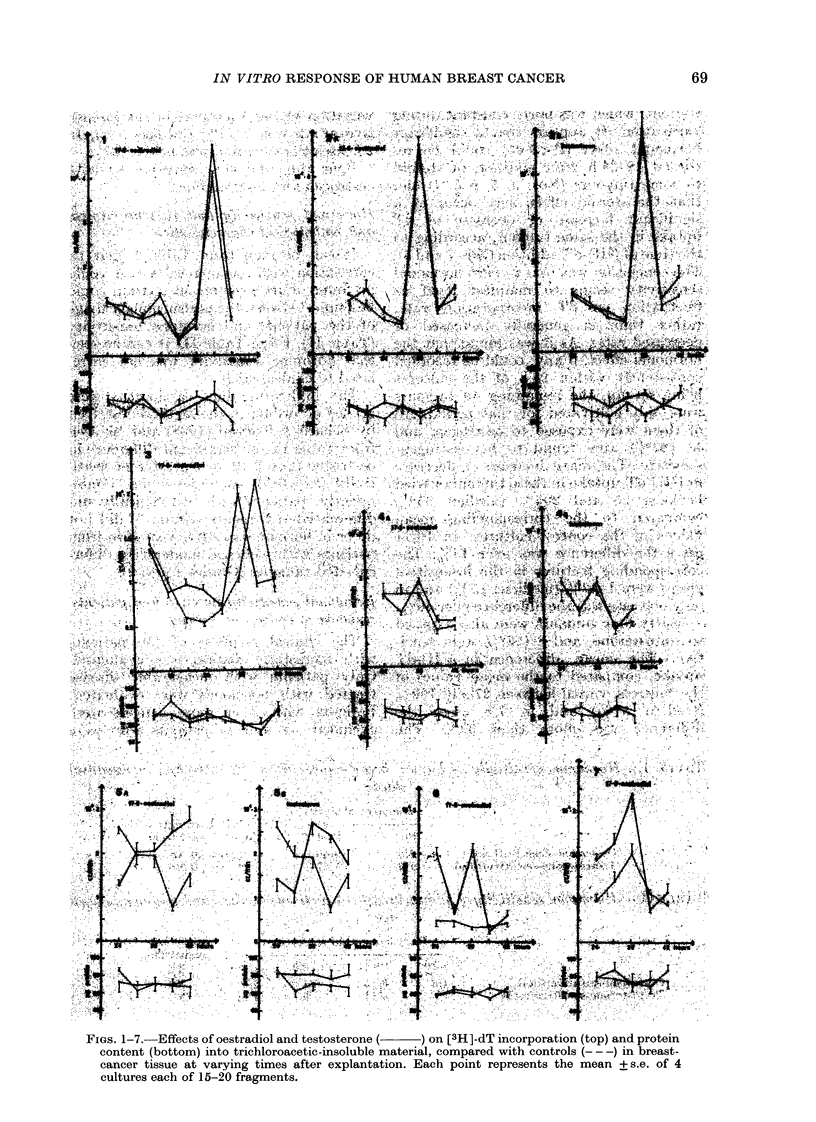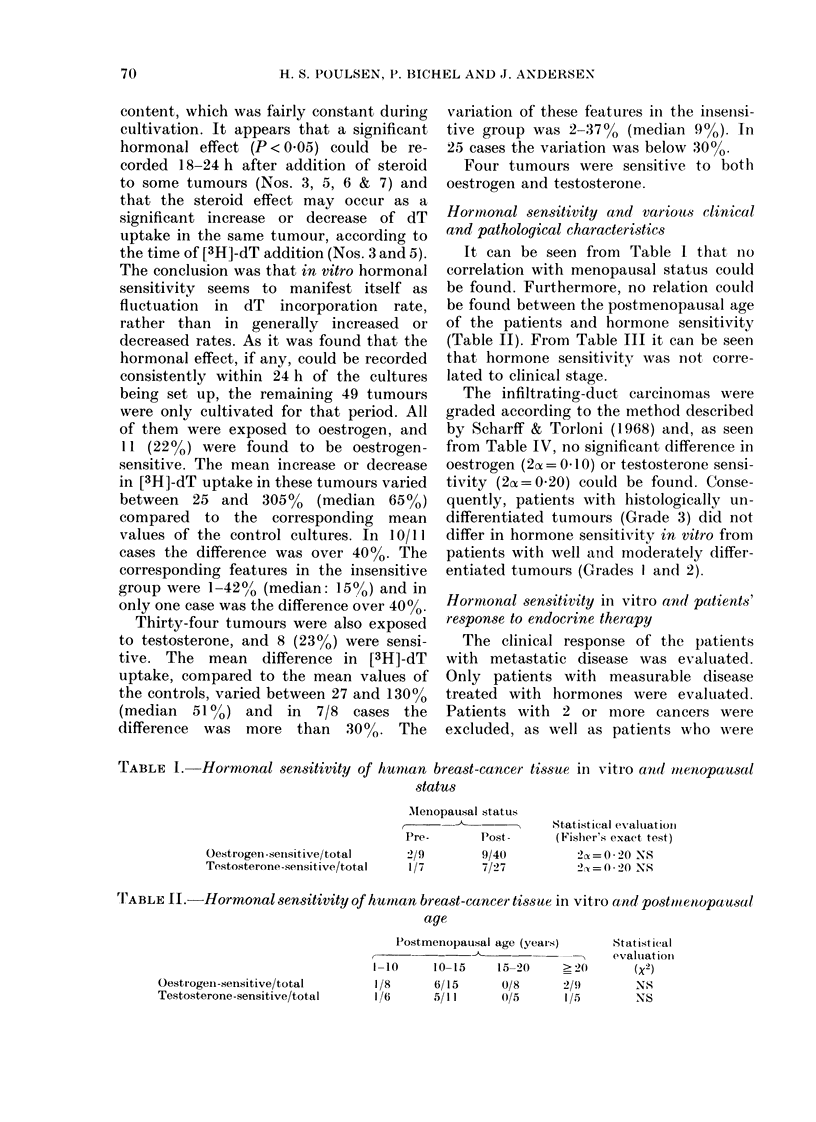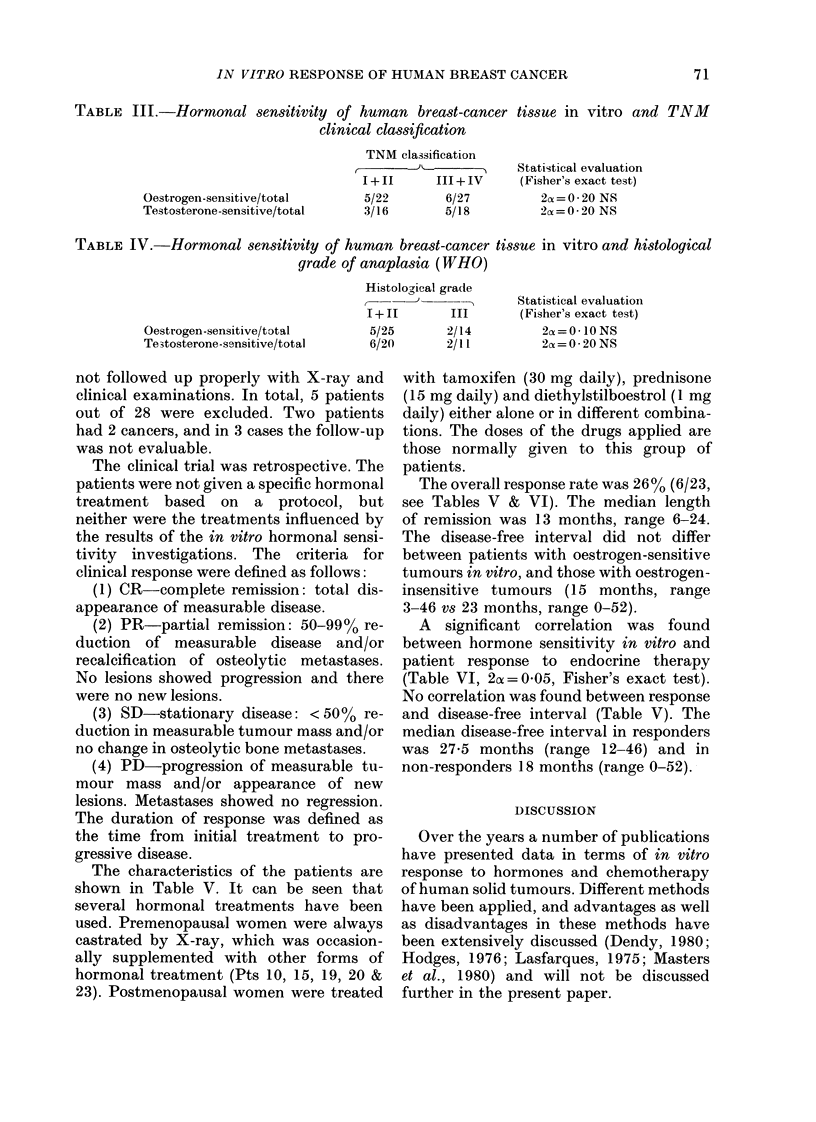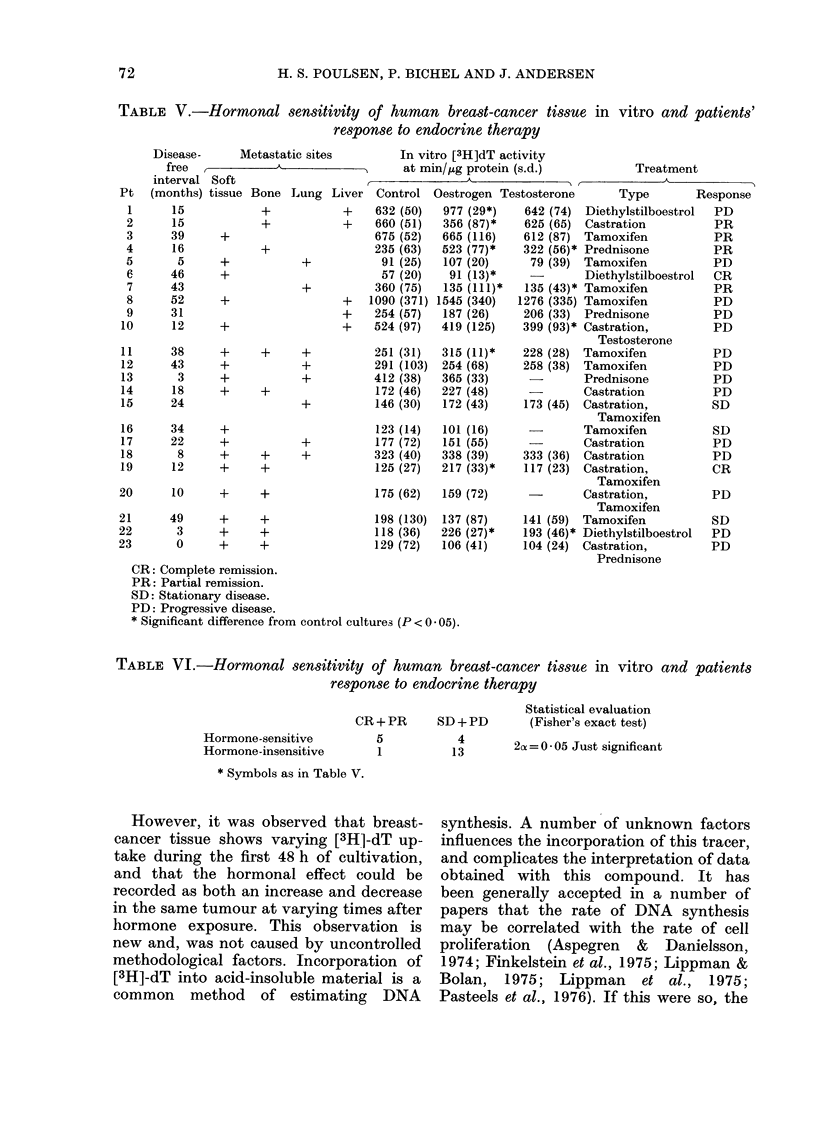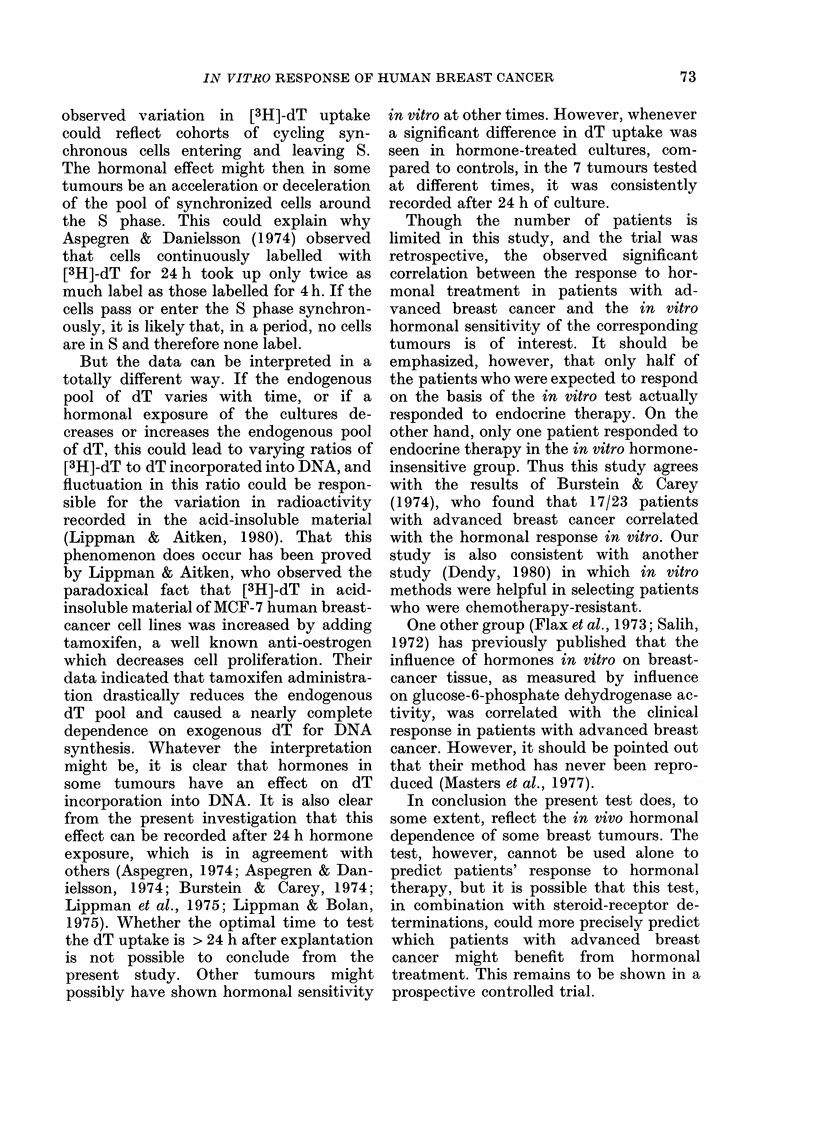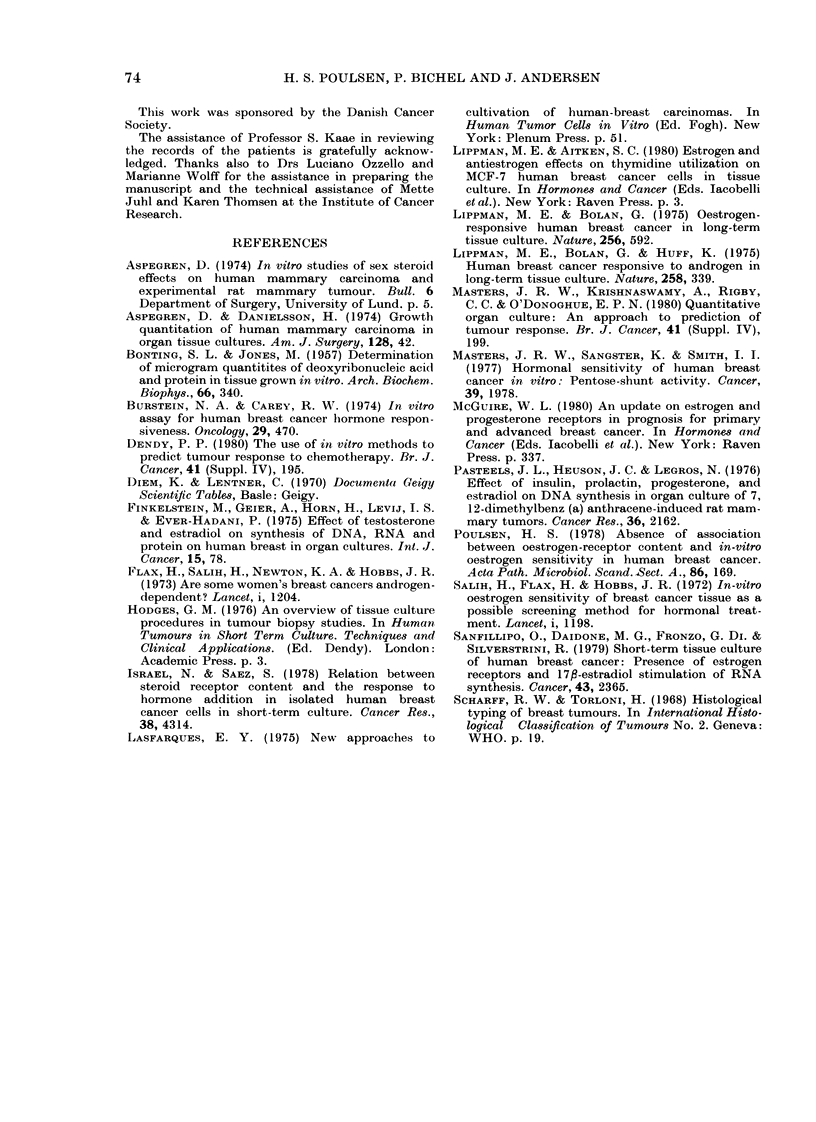# Short-term culture of human breast cancer: in vitro effects of hormones related to patient response.

**DOI:** 10.1038/bjc.1982.166

**Published:** 1982-07

**Authors:** H. S. Poulsen, P. Bichel, J. Andersen

## Abstract

Breast-cancer tissue from 60 patients was tested for oestrogen and testosterone sensitivity in vitro by measuring [3H]-dT incorporation in tissue fragments at various times during 48h culture. Hormone sensitivity in test culture was determined as an increase or decrease in dT uptake. In vitro cultures of breast cancer tissue demonstrate that some tumours are hormone-sensitive and others hormone-insensitive, but it cannot be predicted whether cell proliferation is stimulated or inhibited by hormone treatment. The data were related to the clinical stage of the patients, menopausal status, and the degree of anaplasia of the tumours tested. No correlation was observed between these parameters and in vitro hormonal sensitivity. However, when related to the response of patients to various kinds of hormonal treatment, a significant positive correlation was obtained.


*Br. J. Cancer* (1982) **46**, 67

### SHORT-TERM CULTURE OF HUMAN BREAST CANCER: *IN VITRO* EFFECTS OF HORMONES RELATED TO PATIENT RESPONSE

H. S. POULSEN*†§, P. BICHEL† AND J. ANDERSEN‡

*From the *†Institute of Cancer Research, Radiumstationen, *†University Institute of Pathology and *Department of Surgery, Aarhus County Hospital, DK-8000 Aarhus C, and ‡The Institute of Pathology, Esbjerg Centralsygehus, DK-6700 Esbjerg, Denmark*

Received 23 September 1981 Accepted 2 March 1982

**Summary.—Breast-cancer tissue from 60 patients was tested for oestrogen and testosterone sensitivity *in vitro* by measuring [$^3$H]-dT incorporation in tissue fragments at various times during 48h culture. Hormone sensitivity in test culture was determined as an increase or decrease in dT uptake. *In vitro* cultures of breast-cancer tissue demonstrate that some tumours are hormone-sensitive and others hormone-insensitive, but it cannot be predicted whether cell proliferation is stimulated or inhibited by hormone treatment.**

**The data were related to the clinical stage of the patients, menopausal status, and the degree of anaplasia of the tumours tested. No correlation was observed between these parameters and *in vitro* hormonal sensitivity. However, when related to the response of patients to various kinds of hormonal treatment, a significant positive correlation was obtained.**

ONE THIRD OF PATIENTS with advanced breast cancer respond to endocrine therapy.

The oestrogen-receptor (RE) assay on breast-cancer tissue has been used to select patients for hormonal treatment, but as present only half the patients with RE$^+$ tumours actually benefit from endocrine therapy. It has been shown, however, that tumours containing both RE and progesterone receptors, and tumours containing high RE levels, are more likely to respond (70–80%) than tumours with low RE levels or none (5–10%; McGuire, 1980).

Instead of measuring the initial binding step in the hormone action, another approach would be to determine hormonal influence on the tumours *in vitro*. It has been shown that the *in vitro* effect of hormones on breast-cancer tissue is not simply a reflection of the presence or absence of steroid receptors (Israel & Saez, 1978; Poulsen, 1978; Sanfillipo *et al.*, 1979) and our proposed line of investigation might yield new information.

The purpose of this study was to observe the effect of various hormones on the [$^3$H]-dT incorporation in breast-cancer tissue *in vitro*, and to compare the results with the effect of hormonal treatment on patients with advanced breast cancer.

### MATERIALS AND METHODS

In vitro *technique*.—Radioactive thymidine [$^3$H]-dT was obtained from The Radiochemical Centre, Amersham. Steroids were obtained from Sigma Chemical Co., and foetal calf serum from Flow Laboratories. Insta-gel was obtained from Packard and K4 emulsion from Ilford.

Surgically excised breast-cancer tissue

§ To whom request for reprints should be addressed: at the Institute of Cancer Research, Radiumstationen, 44 Norrebrogade, DK-8000 Aarhus C, Denmark.

* Present address: College of Physicians and Surgeons of Columbia University Surgical Pathology, 630 West 1968th Street, New York, N.Y. 10032.

from 60 patients with primary breast cancer were placed in ice-cold Eagle's minimal essential medium (MEM) and prepared for cultivation within 1 h of removal. All damaged and fatty tissue was removed, and the rest was minced into small fragments ($\sim 0{\cdot}5$ mm$^3$) with a pair of scissors, all under sterile conditions. With a spatula, 15–20 fragments were then randomly transferred to 10 ml test tubes containing 5 ml culture medium and allowed to float freely. The medium was MEM with added antibiotics (100 iu/ml of penicillin and 100 $\mu$g/ml streptomycin) and supplemented with foetal calf serum to a concentration of 5%. Four cultures of 15–20 fragments each were set up for each hormone and for control. All cultures were grown in an atmosphere of 95% humidified air and 5% $CO_2$ at 37°C. The tissue fragments were cultivated under the following conditions; (1) controls, 0·1% ethanol added, (2) 17-$\beta$-oestradiol, 1 $\mu$M, (3) testosterone, 1 $\mu$M.

The steroid hormones were dissolved in ethanol, of which the final concentration was 0·1% of the total volume in the culture medium. They were added to the medium when the cultures were set up.

At different times after explantation, [$^3$H]-dT was added to the cultures for 1 h (2·5 $\mu$Ci/ml, 2·0 Ci/mmol) and the incorporation was terminated by placing the cultures on ice. The supernatant was removed and 5% trichloroacetic acid added for 45 min. After centrifugation (800 *g*, 4°C) for 10 min, the final sediment was dissolved in 2·0 ml 1 M NaOH for 2 h at 60°C. Each sample was divided into two portions. One portion (200 $\mu$l) was prepared for scintillation counting by mixing it with 12 ml scintillation fluid (Insta-gel). Radioactivity was measured in a Packard TriCarb 3003 spectrophotometer. The quench was checked with channel radio correction. The other portion (100 $\mu$l) was prepared for protein measurement by the method of Lowry.

The results were expressed in terms of ct/min/$\mu$g protein. As the results may not be normally distributed, the results of controls and hormone-treated cultures were compared by means of the nonparametric Mann–Whitney test (Diem & Lentner, 1970). When the results from the test-cultures were significantly different from the control cultures ($P < 0{\cdot}05$) the tumours were defined as hormone-sensitive, the others hormone-insensitive.

*Histological procedure.*—From all biopsy specimens received, one representative piece was taken at once for histological examination, and another 15–20 fragments randomly selected from the cultures at the end of cultivation.

*Autoradiography.*—Cultivated tissue fragments were fixed and embedded in paraffin. Three-4$\mu$m sections were placed on gelatinized slides and dipped into Ilford K4 emulsion. The autoradiographs were developed after 1–2 weeks' exposure in lightproof boxes at 4°C. The developed slides were stained with haematoxylin and eosin and evaluated for localization of [$^3$H]-dT in the tissue fragments.

### RESULTS

### *Histological and autoradiographic studies*

The biopsy specimens received were all confirmed as breast cancer. Examination of the cultivated fragments showed that the tissue was well preserved. In general, necrobiosis was only slightly accentuated during cultivation.

In most of the autoradiographs, the background radiation was negligible. The radioactivity was localized in the cell nuclei. Apart from a few fibroblasts, all labelled cells were epithelial. Cells with pyknotic nuclei did not incorporate thymidine.

### *Thymidine incorporation in cultures at varying times after explantation*

Preliminary experiments were carried out to determine culture conditions, and to determine whether specimens from the same tumour showed comparable uptake of [$^3$H]-dT under the same conditions. It was found in 4 tumours that samples consisting of 4 cultures of 15–20 fragments each were comparable, whether protein or DNA content was used (Bonting & Jones, 1957) to estimate the amount of tissue. Any differences in uptake of [$^3$H]-dT would thus be due to hormonal effect on the tumour tissue.

Figs. 1–7 show the results from 7 different tumours. It appears that the dT uptake varies markedly during cultivation, and is not due to varying protein

FIGS. 1–7.—Effects of oestradiol and testosterone (———) on $[^3H]$-dT incorporation (top) and protein content (bottom) into trichloroacetic-insoluble material, compared with controls (– – –) in breast-cancer tissue at varying times after explantation. Each point represents the mean ± s.e. of 4 cultures each of 15–20 fragments.

content, which was fairly constant during cultivation. It appears that a significant hormonal effect ($P < 0.05$) could be recorded 18–24 h after addition of steroid to some tumours (Nos. 3, 5, 6 & 7) and that the steroid effect may occur as a significant increase or decrease of dT uptake in the same tumour, according to the time of [$^3$H]-dT addition (Nos. 3 and 5). The conclusion was that *in vitro* hormonal sensitivity seems to manifest itself as fluctuation in dT incorporation rate, rather than in generally increased or decreased rates. As it was found that the hormonal effect, if any, could be recorded consistently within 24 h of the cultures being set up, the remaining 49 tumours were only cultivated for that period. All of them were exposed to oestrogen, and 11 (22%) were found to be oestrogen-sensitive. The mean increase or decrease in [$^3$H]-dT uptake in these tumours varied between 25 and 305% (median 65%) compared to the corresponding mean values of the control cultures. In 10/11 cases the difference was over 40%. The corresponding features in the insensitive group were 1–42% (median: 15%) and in only one case was the difference over 40%.

Thirty-four tumours were also exposed to testosterone, and 8 (23%) were sensitive. The mean difference in [$^3$H]-dT uptake, compared to the mean values of the controls, varied between 27 and 130% (median 51%) and in 7/8 cases the difference was more than 30%. The variation of these features in the insensitive group was 2–37% (median 9%). In 25 cases the variation was below 30%.

Four tumours were sensitive to both oestrogen and testosterone.

### *Hormonal sensitivity and various clinical and pathological characteristics*

It can be seen from Table I that no correlation with menopausal status could be found. Furthermore, no relation could be found between the postmenopausal age of the patients and hormone sensitivity (Table II). From Table III it can be seen that hormone sensitivity was not correlated to clinical stage.

The infiltrating-duct carcinomas were graded according to the method described by Scharff & Torloni (1968) and, as seen from Table IV, no significant difference in oestrogen ($2\alpha = 0.10$) or testosterone sensitivity ($2\alpha = 0.20$) could be found. Consequently, patients with histologically undifferentiated tumours (Grade 3) did not differ in hormone sensitivity *in vitro* from patients with well and moderately differentiated tumours (Grades 1 and 2).

### *Hormonal sensitivity* in vitro *and patients' response to endocrine therapy*

The clinical response of the patients with metastatic disease was evaluated. Only patients with measurable disease treated with hormones were evaluated. Patients with 2 or more cancers were excluded, as well as patients who were

TABLE I.—*Hormonal sensitivity of human breast-cancer tissue* in vitro *and menopausal status*

| | Menopausal status | | Statistical evaluation |
|---|---|---|---|
| | Pre- | Post- | (Fisher's exact test) |
| Oestrogen-sensitive/total | 2/9 | 9/40 | $2\alpha = 0.20$ NS |
| Testosterone-sensitive/total | 1/7 | 7/27 | $2\alpha = 0.20$ NS |

TABLE II.—*Hormonal sensitivity of human breast-cancer tissue* in vitro *and postmenopausal age*

| | Postmenopausal age (years) | | | | Statistical evaluation |
|---|---|---|---|---|---|
| | 1–10 | 10–15 | 15–20 | ≧20 | ($\chi^2$) |
| Oestrogen-sensitive/total | 1/8 | 6/15 | 0/8 | 2/9 | NS |
| Testosterone-sensitive/total | 1/6 | 5/11 | 0/5 | 1/5 | NS |

TABLE III.—*Hormonal sensitivity of human breast-cancer tissue* in vitro *and TNM clinical classification*

| | TNM classification | | Statistical evaluation (Fisher's exact test) |
|---|---|---|---|
| | I+II | III+IV | |
| Oestrogen-sensitive/total | 5/22 | 6/27 | $2\alpha=0\cdot20$ NS |
| Testosterone-sensitive/total | 3/16 | 5/18 | $2\alpha=0\cdot20$ NS |

TABLE IV.—*Hormonal sensitivity of human breast-cancer tissue* in vitro *and histological grade of anaplasia (WHO)*

| | Histological grade | | Statistical evaluation (Fisher's exact test) |
|---|---|---|---|
| | I+II | III | |
| Oestrogen-sensitive/total | 5/25 | 2/14 | $2\alpha=0\cdot10$ NS |
| Testosterone-sensitive/total | 6/20 | 2/11 | $2\alpha=0\cdot20$ NS |

not followed up properly with X-ray and clinical examinations. In total, 5 patients out of 28 were excluded. Two patients had 2 cancers, and in 3 cases the follow-up was not evaluable.

The clinical trial was retrospective. The patients were not given a specific hormonal treatment based on a protocol, but neither were the treatments influenced by the results of the *in vitro* hormonal sensitivity investigations. The criteria for clinical response were defined as follows:

(1) CR—complete remission: total disappearance of measurable disease.

(2) PR—partial remission: 50–99% reduction of measurable disease and/or recalcification of osteolytic metastases. No lesions showed progression and there were no new lesions.

(3) SD—stationary disease: <50% reduction in measurable tumour mass and/or no change in osteolytic bone metastases.

(4) PD—progression of measurable tumour mass and/or appearance of new lesions. Metastases showed no regression. The duration of response was defined as the time from initial treatment to progressive disease.

The characteristics of the patients are shown in Table V. It can be seen that several hormonal treatments have been used. Premenopausal women were always castrated by X-ray, which was occasionally supplemented with other forms of hormonal treatment (Pts 10, 15, 19, 20 & 23). Postmenopausal women were treated with tamoxifen (30 mg daily), prednisone (15 mg daily) and diethylstilboestrol (1 mg daily) either alone or in different combinations. The doses of the drugs applied are those normally given to this group of patients.

The overall response rate was 26% (6/23, see Tables V & VI). The median length of remission was 13 months, range 6–24. The disease-free interval did not differ between patients with oestrogen-sensitive tumours *in vitro*, and those with oestrogen-insensitive tumours (15 months, range 3–46 *vs* 23 months, range 0–52).

A significant correlation was found between hormone sensitivity *in vitro* and patient response to endocrine therapy (Table VI, $2\alpha=0{\cdot}05$, Fisher's exact test). No correlation was found between response and disease-free interval (Table V). The median disease-free interval in responders was 27·5 months (range 12–46) and in non-responders 18 months (range 0–52).

### DISCUSSION

Over the years a number of publications have presented data in terms of *in vitro* response to hormones and chemotherapy of human solid tumours. Different methods have been applied, and advantages as well as disadvantages in these methods have been extensively discussed (Dendy, 1980; Hodges, 1976; Lasfarques, 1975; Masters *et al.*, 1980) and will not be discussed further in the present paper.

TABLE V.—*Hormonal sensitivity of human breast-cancer tissue* in vitro *and patients' response to endocrine therapy*

| Pt | Disease-free interval (months) | Metastatic sites: Soft tissue | Bone | Lung | Liver | In vitro [$^3$H]dT activity at min/$\mu$g protein (s.d.): Control | Oestrogen | Testosterone | Treatment: Type | Response |
|---|---|---|---|---|---|---|---|---|---|---|
| 1 | 15 | | + | | + | 632 (50) | 977 (29*) | 642 (74) | Diethylstilboestrol | PD |
| 2 | 15 | | + | | + | 660 (51) | 356 (87)* | 625 (65) | Castration | PR |
| 3 | 39 | + | | | | 675 (52) | 665 (116) | 612 (87) | Tamoxifen | PR |
| 4 | 16 | | + | | | 235 (63) | 523 (77)* | 322 (56)* | Prednisone | PR |
| 5 | 5 | + | | + | | 91 (25) | 107 (20) | 79 (39) | Tamoxifen | PD |
| 6 | 46 | + | | | | 57 (20) | 91 (13)* | — | Diethylstilboestrol | CR |
| 7 | 43 | | | + | | 360 (75) | 135 (111)* | 135 (43)* | Tamoxifen | PR |
| 8 | 52 | + | | | + | 1090 (371) | 1545 (340) | 1276 (335) | Tamoxifen | PD |
| 9 | 31 | | | | + | 254 (57) | 187 (26) | 206 (33) | Prednisone | PD |
| 10 | 12 | + | | | + | 524 (97) | 419 (125) | 399 (93)* | Castration, Testosterone | PD |
| 11 | 38 | + | + | + | | 251 (31) | 315 (11)* | 228 (28) | Tamoxifen | PD |
| 12 | 43 | + | | + | | 291 (103) | 254 (68) | 258 (38) | Tamoxifen | PD |
| 13 | 3 | + | | + | | 412 (38) | 365 (33) | — | Prednisone | PD |
| 14 | 18 | + | + | | | 172 (46) | 227 (48) | — | Castration | PD |
| 15 | 24 | | | + | | 146 (30) | 172 (43) | 173 (45) | Castration, Tamoxifen | SD |
| 16 | 34 | + | | | | 123 (14) | 101 (16) | — | Tamoxifen | SD |
| 17 | 22 | + | | + | | 177 (72) | 151 (55) | — | Castration | PD |
| 18 | 8 | + | + | + | | 323 (40) | 338 (39) | 333 (36) | Castration | PD |
| 19 | 12 | + | + | | | 125 (27) | 217 (33)* | 117 (23) | Castration, Tamoxifen | CR |
| 20 | 10 | + | + | | | 175 (62) | 159 (72) | — | Castration, Tamoxifen | PD |
| 21 | 49 | + | + | | | 198 (130) | 137 (87) | 141 (59) | Tamoxifen | SD |
| 22 | 3 | + | + | | | 118 (36) | 226 (27)* | 193 (46)* | Diethylstilboestrol | PD |
| 23 | 0 | + | + | | | 129 (72) | 106 (41) | 104 (24) | Castration, Prednisone | PD |

CR: Complete remission.
PR: Partial remission.
SD: Stationary disease.
PD: Progressive disease.
* Significant difference from control cultures ($P < 0 \cdot 05$).

TABLE VI.—*Hormonal sensitivity of human breast-cancer tissue* in vitro *and patients response to endocrine therapy*

| | CR+PR | SD+PD | Statistical evaluation (Fisher's exact test) |
|---|---|---|---|
| Hormone-sensitive | 5 | 4 | $2\alpha = 0 \cdot 05$ Just significant |
| Hormone-insensitive | 1 | 13 | |

* Symbols as in Table V.

However, it was observed that breast-cancer tissue shows varying [$^3$H]-dT uptake during the first 48 h of cultivation, and that the hormonal effect could be recorded as both an increase and decrease in the same tumour at varying times after hormone exposure. This observation is new and, was not caused by uncontrolled methodological factors. Incorporation of [$^3$H]-dT into acid-insoluble material is a common method of estimating DNA synthesis. A number of unknown factors influences the incorporation of this tracer, and complicates the interpretation of data obtained with this compound. It has been generally accepted in a number of papers that the rate of DNA synthesis may be correlated with the rate of cell proliferation (Aspegren & Danielsson, 1974; Finkelstein *et al.*, 1975; Lippman & Bolan, 1975; Lippman *et al.*, 1975; Pasteels *et al.*, 1976). If this were so, the observed variation in [$^3$H]-dT uptake could reflect cohorts of cycling synchronous cells entering and leaving S. The hormonal effect might then in some tumours be an acceleration or deceleration of the pool of synchronized cells around the S phase. This could explain why Aspegren & Danielsson (1974) observed that cells continuously labelled with [$^3$H]-dT for 24 h took up only twice as much label as those labelled for 4 h. If the cells pass or enter the S phase synchronously, it is likely that, in a period, no cells are in S and therefore none label.

But the data can be interpreted in a totally different way. If the endogenous pool of dT varies with time, or if a hormonal exposure of the cultures decreases or increases the endogenous pool of dT, this could lead to varying ratios of [$^3$H]-dT to dT incorporated into DNA, and fluctuation in this ratio could be responsible for the variation in radioactivity recorded in the acid-insoluble material (Lippman & Aitken, 1980). That this phenomenon does occur has been proved by Lippman & Aitken, who observed the paradoxical fact that [$^3$H]-dT in acid-insoluble material of MCF-7 human breast-cancer cell lines was increased by adding tamoxifen, a well known anti-oestrogen which decreases cell proliferation. Their data indicated that tamoxifen administration drastically reduces the endogenous dT pool and caused a nearly complete dependence on exogenous dT for DNA synthesis. Whatever the interpretation might be, it is clear that hormones in some tumours have an effect on dT incorporation into DNA. It is also clear from the present investigation that this effect can be recorded after 24 h hormone exposure, which is in agreement with others (Aspegren, 1974; Aspegren & Danielsson, 1974; Burstein & Carey, 1974; Lippman *et al.*, 1975; Lippman & Bolan, 1975). Whether the optimal time to test the dT uptake is $> 24$ h after explantation is not possible to conclude from the present study. Other tumours might possibly have shown hormonal sensitivity *in vitro* at other times. However, whenever a significant difference in dT uptake was seen in hormone-treated cultures, compared to controls, in the 7 tumours tested at different times, it was consistently recorded after 24 h of culture.

Though the number of patients is limited in this study, and the trial was retrospective, the observed significant correlation between the response to hormonal treatment in patients with advanced breast cancer and the *in vitro* hormonal sensitivity of the corresponding tumours is of interest. It should be emphasized, however, that only half of the patients who were expected to respond on the basis of the *in vitro* test actually responded to endocrine therapy. On the other hand, only one patient responded to endocrine therapy in the *in vitro* hormone-insensitive group. Thus this study agrees with the results of Burstein & Carey (1974), who found that 17/23 patients with advanced breast cancer correlated with the hormonal response *in vitro*. Our study is also consistent with another study (Dendy, 1980) in which *in vitro* methods were helpful in selecting patients who were chemotherapy-resistant.

One other group (Flax *et al.*, 1973; Salih, 1972) has previously published that the influence of hormones *in vitro* on breast-cancer tissue, as measured by influence on glucose-6-phosphate dehydrogenase activity, was correlated with the clinical response in patients with advanced breast cancer. However, it should be pointed out that their method has never been reproduced (Masters *et al.*, 1977).

In conclusion the present test does, to some extent, reflect the *in vivo* hormonal dependence of some breast tumours. The test, however, cannot be used alone to predict patients' response to hormonal therapy, but it is possible that this test, in combination with steroid-receptor determinations, could more precisely predict which patients with advanced breast cancer might benefit from hormonal treatment. This remains to be shown in a prospective controlled trial.

This work was sponsored by the Danish Cancer Society.

The assistance of Professor S. Kaae in reviewing the records of the patients is gratefully acknowledged. Thanks also to Drs Luciano Ozzello and Marianne Wolff for the assistance in preparing the manuscript and the technical assistance of Mette Juhl and Karen Thomsen at the Institute of Cancer Research.

### REFERENCES

ASPEGREN, D. (1974) *In vitro* studies of sex steroid effects on human mammary carcinoma and experimental rat mammary tumour. *Bull.* **6** Department of Surgery, University of Lund. p. 5.

ASPEGREN, D. & DANIELSSON, H. (1974) Growth quantitation of human mammary carcinoma in organ tissue cultures. *Am. J. Surgery*, **128**, 42.

BONTING, S. L. & JONES, M. (1957) Determination of microgram quantitites of deoxyribonucleic acid and protein in tissue grown *in vitro*. *Arch. Biochem. Biophys.*, **66**, 340.

BURSTEIN, N. A. & CAREY, R. W. (1974) *In vitro* assay for human breast cancer hormone responsiveness. *Oncology*, **29**, 470.

DENDY, P. P. (1980) The use of *in vitro* methods to predict tumour response to chemotherapy. *Br. J. Cancer*, **41** (Suppl. IV), 195.

DIEM, K. & LENTNER, C. (1970) *Documenta Geigy Scientific Tables*, Basle: Geigy.

FINKELSTEIN, M., GEIER, A., HORN, H., LEVIJ, I. S. & EVER-HADANI, P. (1975) Effect of testosterone and estradiol on synthesis of DNA, RNA and protein on human breast in organ cultures. *Int. J. Cancer*, **15**, 78.

FLAX, H., SALIH, H., NEWTON, K. A. & HOBBS, J. R. (1973) Are some women's breast cancers androgen-dependent? *Lancet*, i, 1204.

HODGES, G. M. (1976) An overview of tissue culture procedures in tumour biopsy studies. In *Human Tumours in Short Term Culture. Techniques and Clinical Applications.* (Ed. Dendy). London: Academic Press. p. 3.

ISRAEL, N. & SAEZ, S. (1978) Relation between steroid receptor content and the response to hormone addition in isolated human breast cancer cells in short-term culture. *Cancer Res.*, **38**, 4314.

LASFARQUES, E. Y. (1975) New approaches to cultivation of human-breast carcinomas. In *Human Tumor Cells in Vitro* (Ed. Fogh). New York: Plenum Press. p. 51.

LIPPMAN, M. E. & AITKEN, S. C. (1980) Estrogen and antiestrogen effects on thymidine utilization on MCF-7 human breast cancer cells in tissue culture. In *Hormones and Cancer* (Eds. Iacobelli *et al.*). New York: Raven Press. p. 3.

LIPPMAN, M. E. & BOLAN, G. (1975) Oestrogen-responsive human breast cancer in long-term tissue culture. *Nature*, **256**, 592.

LIPPMAN, M. E., BOLAN, G. & HUFF, K. (1975) Human breast cancer responsive to androgen in long-term tissue culture. *Nature*, **258**, 339.

MASTERS, J. R. W., KRISHNASWAMY, A., RIGBY, C. C. & O'DONOGHUE, E. P. N. (1980) Quantitative organ culture: An approach to prediction of tumour response. *Br. J. Cancer*, **41** (Suppl. IV), 199.

MASTERS, J. R. W., SANGSTER, K. & SMITH, I. I. (1977) Hormonal sensitivity of human breast cancer *in vitro*: Pentose-shunt activity. *Cancer*, **39**, 1978.

MCGUIRE, W. L. (1980) An update on estrogen and progesterone receptors in prognosis for primary and advanced breast cancer. In *Hormones and Cancer* (Eds. Iacobelli *et al.*). New York: Raven Press. p. 337.

PASTEELS, J. L., HEUSON, J. C. & LEGROS, N. (1976) Effect of insulin, prolactin, progesterone, and estradiol on DNA synthesis in organ culture of 7, 12-dimethylbenz (a) anthracene-induced rat mammary tumors. *Cancer Res.*, **36**, 2162.

POULSEN, H. S. (1978) Absence of association between oestrogen-receptor content and *in-vitro* oestrogen sensitivity in human breast cancer. *Acta Path. Microbiol. Scand. Sect. A.*, **86**, 169.

SALIH, H., FLAX, H. & HOBBS, J. R. (1972) *In-vitro* oestrogen sensitivity of breast cancer tissue as a possible screening method for hormonal treatment. *Lancet*, i, 1198.

SANFILLIPO, O., DAIDONE, M. G., FRONZO, G. DI. & SILVERSTRINI, R. (1979) Short-term tissue culture of human breast cancer: Presence of estrogen receptors and 17$\beta$-estradiol stimulation of RNA synthesis. *Cancer*, **43**, 2365.

SCHARFF, R. W. & TORLONI, H. (1968) Histological typing of breast tumours. In *International Histological Classification of Tumours* No. 2. Geneva: WHO. p. 19.